# Advancing Regulatory Oversight of Medical Device Trials to Align with Clinical Drug Standards in the European Union

**DOI:** 10.3390/ph18060876

**Published:** 2025-06-12

**Authors:** Ádám Pannonhalmi, Bence Sipos, Róbert Imre Kurucz, Gábor Katona, Lajos Kemény, Ildikó Csóka

**Affiliations:** 1 Department of Dermatology and Allergology, University of Szeged, Korányi Alley 6, H-6720 Szeged, Hungary; pannonhadam@gmail.com (Á.P.); kemeny.lajos@med.u-szeged.hu (L.K.); 2 Institute of Pharmaceutical Technology and Regulatory Affairs, University of Szeged, Eötvös Street 6, H-6720 Szeged, Hungary; katona.gabor@szte.hu (G.K.); csoka.ildiko@szte.hu (I.C.); 3 Competence Centre of Pharmaceutical Development and Clinical Trials, Centre of Excellence for Interdisciplinary Research, Development and Innovation, University of Szeged, Dugonics Square 13, H-6720 Szeged, Hungary; kurucz.robert@szte.hu

**Keywords:** medical device, medicinal product, drug development, clinical trial, regulatory science, medical device regulation, clinical trials regulation

## Abstract

The regulation of clinical trials for medicinal products and medical devices has undergone numerous changes in recent years in the European Union, challenging manufacturers and national regulatory agencies as well. With the introduction of combined drug–device products, the regulatory landscape has been drastically changed to adapt to novel technological advancements and innovations. A comparative analysis has not yet been published highlighting the main differences and common elements of these two medicinal products, which took up almost all of the market in the pharmaceutical sector. Due to stricter regulations in the field of medical devices, the process from application up until post-market surveillance became more difficult, but a correlation between the regulation of drug trials can also be found. The main differences lie in the risk management systems, where, regardless of the background knowledge of a drug, it is always strict and mandatory structured progress, while in the case of medical devices, it is more flexible based on the risk category of the product. Generally, the utilization of e-health opportunities, transparency, and data accessibility have been improved in both fields. Via the adaptation of the mentioned regulation in the EU, the safety of patients and the efficacy of trials have been greatly increased. This manuscript aims to compare the specific regulations of these two types of medicinal products with a brief outlook on the non-EU sector as well.

## 1. Introduction

The global market for drugs and medical devices is a rapidly evolving sector of the pharmaceutical industry, directly affecting daily interpretation and utilization of innovations in healthcare, academic education, research, and economic development. This market has a wide spectrum where pharmaceuticals, medical devices, and combined products all play a crucial role posing opportunities for novel medical applications but also posing challenges to various stakeholders, including policymakers, healthcare professionals, manufacturers, and finally, the consumers themselves [1,2].

To achieve patient access to innovation, a fast-paced competition consumes these stakeholders where the awareness of market access barriers and regulatory approvals is a critical time-determining factor. The global market is driven by the development and distribution of cutting-edge pharmaceuticals and medical devices, mostly targeting unmet clinical needs or improving therapeutic options not sufficient for large populations. To achieve patient access to innovation, a fast-paced competition consumes these stakeholders, where the awareness of market access barriers and regulatory approvals is a critical time-determining factor. The global market is driven by the development and distribution of cutting-edge pharmaceuticals and medical devices, mostly targeting unmet clinical needs or improving therapeutic options that are insufficient for large populations. Unmet clinical devices are derived from numerous global health policy agencies, such as the World Health Organization’s list of top 10 unmet clinical needs. Financial databases also provide insight, such as Globaldata, which summarizes and follows the trends of drug and medical device market for specific diseases, epidemiological data and its financial revenue to various companies [3,4]. The European regulatory frameworks define unmet clinical needs as medical conditions which lack authorized diagnostic or preventive or therapeutic methods within the EU or have insufficient current treatment options to effectively manage diseases according to EMA orphan designation and PRIME guidelines [5,6,7]. The diverse nature of the products also indicates the extent of work and time that innovators spend on the research and development (R&D) of each product [8,9]. The highly concentrated intellectual property of big pharma players also poses a challenge to small manufacturers where some of the novel products would not meet the market due to the lack of commercialization resources or the capital behind that enterprise [10,11].

The influence of emerging technologies also plays a crucial role in the market entry of pharmaceuticals and medical devices. Many advancements in artificial intelligence, biotechnology—one of the most researched areas in this sector—and digital health transform and skyrocket drug development and medical device innovation. This not only limits the efforts required to choose the next potential blockbuster active substance or device but fast tracks the innovation as well [12,13]. To ensure successful adaptation and market growth the understanding of novel technologies is of paramount importance. One area of this is the utilization of digital health tools, such as telemedicine or wearable devices which reshape patient care and the market adaptation of prior therapeutic options [14,15,16,17].

The main burden in evaluating the safety, efficacy, and quality of drugs and medical devices lies in the clinical trials before they receive regulatory approvals. The trials must follow a rigorous, multi-phase process to assess therapeutic benefits and identify potential adverse effects regarding dosing, administration route, etc. Robust clinical data is required to ensure that the investigational products meet stringent efficacy and safety requirements before their introduction into clinical practice. In contribution to personalized medicine, a broad spectrum of data must be collected regarding drug/device interactions, and population-specified therapeutic responses. As they are an indispensable part of medical innovation in the pharma sector, the understanding of regulatory requirements and adherence to them is the first step in every R&D process [18,19].

This review aims to identify and summarize the differences between the requirements of clinical trials for drugs, medical devices, or drug–device products. The review focuses on the analysis of the exigencies of the European Union’s Clinical Trials Regulation for Medicinal Products and the Medical Device Regulation. The methodology to evaluate drug and medical device clinical trial differences and similarities requires analyzing the governing regulations through a comparative study of EU Regulation (EU) No 536/2014 for drugs and EU Regulation (EU) 2017/745 for medical devices by examining trial design and approval processes and ethical oversight and transparency requirements. The analysis relies on process mapping to show procedural steps and regulatory actors and selected case studies from public databases (e.g., EudraCT, EUDAMED) to identify practical differences in trial implementation. The analysis draws additional insights from academic literature and regulatory reports and stakeholder perspectives when possible. The analysis aims to reveal regulatory convergence areas while explaining sector-specific limitations and adaptable elements.

## 2. Challenges in the Research and Development of Medicinal Products (Drugs)

As drug R&D is a complex, highly regulated, and resource-intensive process with a longer and more diverse history compared to medical devices, multiple national and international guidelines have been placed into action. Regardless of the (inter)national regulations, common challenges can be found regarding the harmonization and execution of clinical trials. Generally, drug development is an expensive process, especially for innovations where manufacturing is highly costly compared to others, such as for biologics compared to small-molecular-weight molecular drugs, where the production can be simplified, well-determined, and reproducible [20,21,22]. The Alliance for Regenerative Medicine (ARM) published a 2023 report showing that the proposed Joint Clinical Assessment methodology would have rejected 90% of Advanced Therapy Medicinal Products (ATMPs) currently authorized in the EU, thus demonstrating the challenging regulatory framework SMEs face [23,24]. The European Federation of Pharmaceutical Industries and Associations (EFPIA) has highlighted the financial risks faced by SMEs because drug development costs between EUR 290 million and EUR 2.6 billion create substantial challenges for these companies. Small- and medium-sized enterprises (SMEs) face higher risks from clinical trial failures because they do not have diversified pipelines or substantial financial reserves like larger firms do [25,26]. On the other hand, non-biological complex drugs, such as biologics, and nanotechnology-based novel drug delivery systems are often still irreproducible, and their analytical evaluation is also harder. In all cases, clinical trials contribute to a great financial burden, especially for candidates that fail during the process. This high risk can be only acknowledged by larger enterprises, whereas small- and medium-sized enterprises might not take up the challenge due to the potential of failure [27,28,29].

Time is also a critical factor in drug development. On average, it takes 10 to 15 years from discovery to market approval, and this can be extended based on the required clinical data from the clinical trials. Meeting stringent safety and efficacy requirements can also extend study durations. The other prolonging factor could be long and high resource-required regulatory approval processes, especially where the market strategy includes commercialization internationally. Before approval, regulatory agencies require extensive (pre)clinical data in compliance with the evolving regulatory frameworks and the region-by-region varying global standards [30,31,32]. Post-market surveillance and pharmacovigilance as monitoring duties also require a prolonged period to ensure long-term safety as another burden to the regulations. The rise in adverse events after approval leads to drug recalls and regulatory restrictions also placing the market under high supervision and strict regulations. As such, companies must be responsible for an efficient and robust pharmacovigilance program to monitor the performance of the drug in real time. Unsolved cases or those not responding to pharmacovigilance events can also lead to legal liabilities such as lawsuits and financial penalties [33,34]. The European Union’s trastuzumab (Herceptin) biosimilars case demonstrates the patent thickets’ complex nature. The drug faced multiple secondary patents from developers which protected different aspects of the drug including its formulations and usage methods. Biosimilar manufacturers successfully fought against numerous patents to enter the market despite these challenges [35,36]. The European Commission’s 2009 Pharmaceutical Sector Inquiry revealed strategic patenting practices which included divisional applications to build patent clusters. The identified practices functioned as competitive delay mechanisms which allowed patent holders to maintain market exclusivity longer than their original patent period [37,38]. Intellectual property and the issues of patenting a drug are of paramount importance as they ensure exclusivity to drug developers while incentivizing the invested capital in research and development. Patents last only for a limited amount, typically 20 years starting from the filing date during which time the company can recoup any investment before the entrance of generic competitors into the market. There are multiple strategies, including the creation of patent thickets and evergreening to ensure the extension of market exclusivity. Another aspect of intellectual property is when there is a crisis or public health emergency when governments can issue the compulsory licensing of a drug to allow generic production in the name of the common good [39,40]. This is specifically true in low- and middle-income countries where due to high costs, access to life-saving medicines is limited. Comparing the non-biological complex drugs with the small-molecule drugs, biologics also face complex patent challenges due to their complexity in structure and manufacturing [41,42,43].

## 3. Challenges in the Research and Development of Medical Devices

The Medical Device Regulation (MDR) changed the regulatory framework compared with the prior Medical Device Directive (MDD) [44]. The complete integration from May of 2021 brought significant challenges for medical device manufacturers, developers, and the (inter)national regulators. In this section, a structural demonstration will be assessed of the challenges that have risen. The MDR system has led to the reclassification of numerous devices from Class I or IIa to Class IIb or III, which demands more complex clinical investigations and post-market clinical follow-up (PMCF). Under MDR, all active implantable devices and their accessories are now classified as Class III, the highest risk category, necessitating rigorous clinical evaluations and conformity assessments. Devices composed of substances intended to be introduced into the human body via a body orifice or applied to the skin have been up-classified [45]. For instance, certain nasal sprays and wound protection creams, previously Class I, may now fall under Class IIa or IIb, depending on their absorption and systemic effects. The transition has caused longer trial periods because of enhanced sample size needs and more demanding endpoints, and longer follow-up durations. The new requirements for clinical evidence and technical documentation have forced SMEs and manufacturers of all sizes to dedicate major financial and human resources [46]. The process has proven especially challenging for existing MDD devices because they need new conformity assessments, together with updated clinical data, to stay in the market. The European Commission’s MDCG and MedTech Europe have documented product recertification delays and device withdrawals, and notified body capacity shortages, which demonstrate MDR implementation’s extensive system-wide effects. The revised manuscript includes these points along with supporting references to demonstrate how MDR reclassification affects clinical development timelines and strategic planning for higher-risk device categories [45]. However, as standards of current therapeutic protocols, most available devices would be unchanged in terms of marketing; in-development phase products and novel products now require more rigorous evaluation and clinical evidence. Thus, not only the complexity of documentation was changed but also the increased data requirements from the clinical trials making them costly. This led to the narrowing of medical device manufacturers, as smaller companies without adequate financial resources lost their competitiveness. Detailed information should be incorporated most strictly into the application for authorization. The manufacturers must provide a heavily detailed description of the device, the intended purpose, indications, contraindications, and the clinical benefits. If there are any specifics related to the developed device, such as technical characteristics, functional specifications, and device operational technicalities, they should also be included. As a general requirement, the manufacturing processes, design verification, and validation must also be included.

The Medical Device Coordination Group (MDCG) oversees all aspects of medical device development up to market placement as an expert advisory body established under the MDR and Regulation (EU) 2017/746 on in vitro diagnostic medical devices (IVDR). As a basic principle, the manufacture of medical devices must fall under the implementation of the Quality Management System (QMS) by Article 10(9) of the MDR. Regulatory compliance is of highest importance, with detailed management tools on resource and personnel management, supplier and subcontractor control, monitoring of product quality and performance, and finally, the risk management implied in the manufacturing stage. According to MDCG 2021-24, risk management is an integral component of QMS and must be implemented according ISO 14971 [47]. As an additional risk of the 21st century, software and cybersecurity threats must also be addressed, guided by MDCG 2019-16 and MDCG 2022-5, to validate the software according to its intended purposes and ensure cybersecurity measures are embedded from design to decommissioning.

Clinical trials of medical devices are also heavily inspected by the MDCG, with additional guidelines such as MDCG 2020-13 and MDCG 2020-5. As mentioned before, the main burden of proof lies in the results of clinical efficacy, whereas a Clinical Evaluation Plan (CEP) and a Clinical Evaluation Report (CER) are required to be submitted. It is a demonstrative description of the device that would be utilized for medical applications, and the safety, efficacy, and quality must be reflected in the positive results of the clinical trials. The MDR has strengthened its expectations for comparative evaluation by requiring manufacturers to demonstrate their selection of equivalent devices through specific criteria (e.g., technical, biological, and clinical characteristics as outlined in MDR Annex XIV). The practice of obtaining detailed clinical or technical data for competitor devices faces a major obstacle because such information remains protected by intellectual property rights or remains undisclosed to the public. The absence of equivalence-based justifications has resulted in increased use of direct clinical investigations for Class III and implantable devices. The absence of harmonized methodologies for comparator selection results in inconsistent CER quality and manufacturer-to-manufacturer variability in CER quality and consistency. The Medical Device Coordination Group (MDCG) has issued MDCG 2020-5 as a guidance document to clarify expectations but there is no established method for evaluating comparator appropriateness especially when data sources are limited. Manufacturers must conduct additional clinical investigations or create post-market clinical follow-up (PMCF) plans to meet notified body requirements which extends development timelines and increases costs. A novel aspect in the authorization process also includes the device comparison to the currently marketed devices, and it could be based on the literature reviews, post-market surveillance, and references to other clinical devices with similar properties. A post-market clinical follow-up (PMCF) plan must also be included [31]. Conducting clinical trials is challenging in the field of medical devices due to the complex regulatory requirements. It is also challenging to define whether a clinical trial is required, as multiple factors must be included in the case of novel medical devices, such as the conformity assessment routes (meaning which class the medical device belongs to). The pace and the required data also differ for each class, for which guidance can be found in the MDCG 2021-24 and 2021-25 advisory guidelines by MDR Annex VIII. In comparison with drug trials, the device trials must also account for the interaction between the user and the device, which is a highly variable factor. Also, the recruitment process can take a long time, especially when the device is aimed at a rare condition or requires surgical implantation. For example, patients who would receive an implanted device are prone to a long-term follow-up program, and the results would not show in an instant, e.g., for mobility assistance devices, where an additional rehabilitation program is required to help the device achieve its purpose. The integration of digital health tools, including remote-monitoring devices and patient apps and telemedicine platforms, represents a key approach to achieve more frequent lower-burden data collection while minimizing the need for in-person visits. The implementation of these tools enhances patient engagement and compliance which leads to decreased loss to follow-up rates. Patient follow-up embedded in routine care pathways together with the use of existing electronic health records (EHRs) or national registries improves long-term data completeness while minimizing duplicate work. The success of long-duration studies depends on patient-centric trial designs which include flexible visit scheduling and transportation support and consistent communication from study coordinators. Real-time data verification tools and centralized monitoring systems enable early detection of inconsistencies which ensures data integrity. The success of long-term follow-up depends on both clinical site agreements for extended participation and investigator incentives that match follow-up targets.

As mentioned before, the main burden of proof lies in the results of the clinical efficacy, whereas a Clinical Evaluation Report (CER) is required to be submitted. It is a demonstrative description of the device that would be utilized for medical applications and the safety, efficacy, and quality must be reflected on the positive results of the clinical trials. A novel aspect in the authorization process also includes the device comparison to the currently marketed devices and it could be based on the literature reviews, post-market surveillance, and references to other clinical devices with similar properties. A post-market clinical follow-up (PMCF) plan must also be included [44]. Conducting clinical trials is challenging in the field of medical devices due to the complex regulatory requirements. In comparison with drug trials, the device trials must also account for the interaction between the user and the device which is a highly variable factor. Also, the recruitment process can take a long time, especially when the device is aimed at a rare condition and requires surgical implantation. For example, patients who would receive an implanted device, are prone to a long-term follow-up program and the results would not show in an instant, e.g., for mobility assistance devices where an additional rehabilitation program is required to help the device’s purpose. Scaling up while maintaining quality and compliance with manufacturing guidelines such as Good Manufacturing Practice (GMP) can also be challenging. To ensure batch consistency, bioequivalence, and stability, the integrity of the supply chain and the competencies of the manufacturers must be in harmony [48,49,50,51,52].

In addition to the supplementary clinical data required, this would also increase the costs of compliance with the regulatory standards from an administrative perspective as well. It is particularly burdensome for small and medium enterprises which may struggle with the financial aspects of meeting the new regulatory requirements. This leads to a high demand for personal and resource assets. The new regulatory standards also limit the notified bodies due to the stricter conditions a body could evaluate the authorization of medical devices. This led to a bottleneck-like system where the manufacturers face longer certification times leading to a delay in their marketing strategy [48,50].

The post-market surveillance for medical devices requires ongoing monitoring concerning device performance after release to the market. Real-world utilization of devices poses challenges and variability in patient populations and environments compared to clinical trials, where a heavily controlled environment is applied to certain patients. These variations can impact previous statements from clinical trials regarding device safety and therapeutic efficacy. Identifying and reporting adverse events is challenging due to the underreporting by healthcare providers or the patients themselves where the adverse effects cannot be connected to the malfunction of the device. Complex data collection and analysis can be eased nowadays due to the technological and IT advances in electronic health records, patient registries, and voluntary reports. However, if the device is placed in multiple countries’ markets, the source of data can differ, and the national data requirements can vary, which would make the comparison of incoming data difficult. The global markets also add a level of complexity, where the different regions and their corresponding bodies may have varying standards and timelines, thereby making comprehensive global surveillance strenuous [53,54,55]. The International Medical Device Regulators Forum (IMDRF) works to establish worldwide PMS frameworks through initiatives that define common terminology and reporting formats and risk classification models. The IMDRF adopted Medical Device Adverse Event (MDAE) terminology and coding system to create uniform reporting standards between member states. National competent authorities can share data through centralized digital platforms which function similarly to the European database on medical devices (EUDAMED) that standardizes vigilance and clinical investigations and PMS data for regulators and manufacturers and public access. The implementation of global platform expansion or regional database alignment to a standardized data model would enhance international consistency while minimizing duplicated work. Regulatory bodies need to establish uniform PMS requirements which include standardized incident reporting schedules and identical signal validation standards and standardized post-market study approaches. The EU and ASEAN have established regulatory science networks to enable mutual recognition of PMS findings through coordinated assessment procedures which reduce the need for duplicate data collection.

Each phase of medical device development is highly controlled by strict regulatory standards and manufacturers must comply with Good Manufacturing Practices (GMP) and ISO standards [56]. This requires the implementation of quality control at multiple levels in scope with the phase the devices are at. The variability of raw and acceptable materials also varies by region, affecting the final product’s consistency and uniformity, which leads to the necessity of stringent supplier management and raw material testing. Novel technologies, especially for intricate designs, also pose the danger of the lack of product uniformity after the production is scaled up where the desired quality may vary batch by batch. A critical point in manufacturing also includes the adaptability to execute changes in the device and/or production technology based on post-market surveillance reports and feedback. The implementation of IT advances in the product itself also requires a more skilled workforce with new positions and competencies in the manufacturer. In compliance with regulatory standards, longer development timelines must be expected as well; in addition, labeling, packaging, and traceability pose challenges for manufacturing, where a unique device identification system is needed. Creating innovation in the industry is also of paramount importance; however, the new strict regulations might create barriers to progress. Potential disincentives can originate from material limitations, as well as shifts in the market that could change the company’s directives and market placement strategies.

Finally, an amplified demand for risk management occurs after the implementation of these mandatory regulation changes for the medical device industry. Ensuring patients’ safety whilst complying with regulatory standards poses a great challenge where manufacturers must identify, evaluate, and most importantly mitigate the potential risks throughout the product lifecycle from the early preclinical development phases until the post-market surveillance. Some standards are determined by the application of GMP and ISO guidelines such as ISO 14971, which requires a detailed risk analysis based on their occurrence and their potential impact on device performance and the users themselves [47]. The International Medical Device Regulators Forum (IMDRF) has developed globally accepted guidance on risk classification and clinical evaluation through IMDRF/GRRP WG/N47 and WG/N56, which provide a basis for regulatory alignment. National authorities who adopt these frameworks in full would create better consistency in risk evaluation for similar device categories. The EU Medical Device Regulation (MDR) Common Specifications (CS) provide structured expectations for device safety and performance in contexts where harmonized standards or clinical evidence remain limited. The specifications have the potential to decrease assessment variability and improve notified body comparison when they are used to assess more device types. The development of device-specific guidance documents together with harmonized classification rules between major regulatory agencies such as EMA, FDA, and PMDA would support alignment for complex or novel technologies like AI-driven software or combination products. The documents could include standardized templates to evaluate benefit–risk profiles, as well as usability and real-world performance. ISO 14971 risk management standards function as a fundamental requirement for international use. The adoption of these standards in regulatory submissions together with mutual recognition of conformity assessments would create a globally consistent and efficient evaluation process which reduces the regulatory burden on manufacturers. The manufacturer’s choice for risk analysis and management can vary, and multiple conventional techniques can be applied such as failure mode analysis, risk–benefit assessment, etc. These techniques also pose the problem of not being uniform; thus, similar products must meet different standards and factors considered despite the same indication. Maintaining functionality in balance with risk reduction is of paramount importance, and sometimes this leads the device manufacturer to make difficult trade-offs, particularly for novel technologies, reducing their complexity and sacrificing uniqueness. Manufacturers must also continuously update risk assessments due to the variation in product performance based on post-market surveillance. The dynamic nature of risk management makes it extremely challenging to maintain consistency. This is especially true for software-driven products, where new versions are released requiring sustained investment. A novel necessity of risk management has also entered the 21st century, namely cybersecurity risk management, where potential threats like hacking and software malfunctions must be addressed. Since a shift towards software-driven devices can be experienced in current research and development trends, this also poses a great danger to the market safety of the device manufacturer [57,58,59].

**Figure 1:**
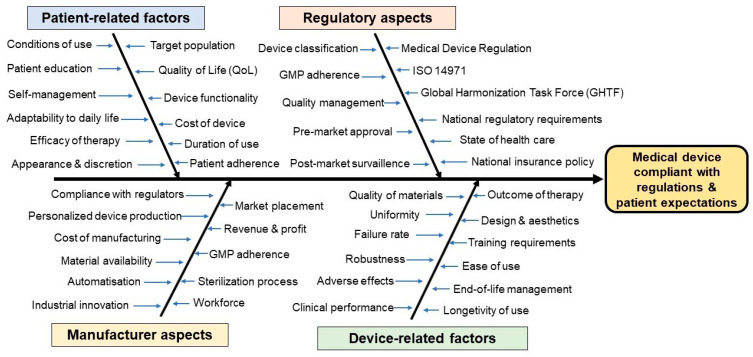
Ishikawa diagram on the main factors affecting the development of medical devices compliant with regulations and patient expectations. Abbreviations used: QoL—Quality of Life; ISO—International Organization for Standardization; GMP—Good Manufacturing Practice; GHTF—Global Harmonization Task Force.

Medical devices incorporating molecular imaging and diagnostic technologies encounter distinct challenges because they unite biological elements with chemical compounds and sophisticated imaging equipment. The fast technological advancements of these devices require regulatory systems to develop new approaches for handling diagnostic–therapeutic boundary issues. The establishment of suitable performance and safety standards proves challenging because these technologies operate through multiple mechanisms across various clinical applications. The complex nature of molecular imaging agents and devices makes it difficult to obtain robust clinical evidence which demands new trial approaches and different endpoints. Real-world evidence and advanced analytics provide opportunities to support regulatory submissions and post-market surveillance. The standardization of international regulatory procedures helps eliminate redundant processes which enables faster patient access to advanced molecular imaging diagnostic tools. The regulatory pathway requires developers to understand preclinical and clinical data requirements through clear classification rules. The regulatory pathway requires essential collaboration between regulators and industry representatives and academic researchers to maintain technological progress. The devices’ generation of sensitive biological information requires strict attention to ethical matters, including patient safety and data privacy, as well as informed consent procedures. The development of evolving regulatory frameworks needs to strike a balance between supporting innovation and conducting strict oversight to provide patients with efficient access to molecular imaging diagnostics [60,61]. As a summary, the factors affecting medical device development and market placement can be found in Figure 1.

## 4. Regulation of Clinical Trials for Drugs

In the EU, Clinical Trials Regulation No. 536/2014 is implemented to govern the conduct of clinical trials involving medicinal products for human use [62]. It was adopted on the 16 April 2014 and made applicable on the 31 January 2022. Prior to that, the Clinical Trials Directive 2001/20/EC was utilized. This particular regulation aims to harmonize the assessment and supervision of all clinical trials executed in the EU member states. It not only ensures the quality of clinical trials but also promotes innovation in medical research with high safety and transparency standards. Elements and mandatory requirements of Good Clinical Practice (GCP, ICH E6) are also included in Regulation 536/2014 [63]. There were multiple discrepancies in the implementation of clinical trials across each member state, leading to heavy administrative burdens and inefficient authorization processes. The regulation aims to create a single, streamlined system to apply for every one of the clinical trial applications, to gain public access to clinical trial data, while ensuring that all participating patients’ safety is met to the highest standard. A comparison of the Clinical Trials Regulation No. 536/2014 and Clinical Trials Directive 2001/20/EC can be found in Table 1.

The key feature of Regulation No. 536/2014 is the implementation of a single clinical trials application and authorization process applied to each member state on a centralized online platform, namely the Clinical Trials Information System (CTIS). It is designed to streamline the submission process, the assessment, and the supervision by regulatory agents of clinical trials across the EU. It is accessible to all stakeholders to ensure that all steps are transparent and efficient, and all data can be unified to the regulatory bodies, the sponsors, and the public. The sponsors submit a single application form via CTIS for clinical trials conducted in multiple member states to avoid the duplication of data. The application can be divided into two main parts. The first part includes the coverage of scientific and clinical aspects, the trial design, applied methodology, what safety measures are applied, and the risk assessment process and evaluation strategy for the resulting data. This process ensures that the study meets high scientific and ethical standards. The second part includes the national-specific considerations based on patient recruitment strategies, specific ethical review processes, and consent procedures to meet the individual member state’s requirements. After all data has been provided in the submission, a harmonized decision-making process follows, involving coordinated assessments [63]. Technical complexity and non-user-friendly design of CTIS have received criticism because they create difficulties for sponsors, especially SMEs and academic institutions, in navigating multinational trials. The system needs substantial training and time commitment before users can master its operation. The validation and assessment processes experience frequent delays because different national competent authorities implement regulatory timelines inconsistently which results in extended and fragmented approval periods. The centralized CTIS platform has shown difficulties in document and version control, especially when managing amendments across multiple jurisdictions. Technical problems with communication features and delayed notification systems have caused confusion and missed deadlines, which negatively impact the efficiency of trial execution [64].

After the submission, an initial validation phase is implemented, where the regulatory authorities check and decide on completeness and compliance with the required standard. Following validation, a review process begins, both scientifically and ethically involving regulatory agencies, relevant public health authorities and, last but not least, the ethics committees. This is a strict process to ensure that the trial is acceptable in the early phases based on the possible risk factors associated. If only one member state is involved in the trial, then the reporting member state is the leader of evaluation, and they provide the initial decision. If other members are involved, then all evaluations and decisions must be collected before the final authorization. If approved, the sponsors and the regulatory bodies both continue to monitor and fill in data on the progress through CTIS. These data include regular updates on trial progress and any protocol modifications and amendments. Reporting adverse and safety-concerning events is mandatory; if missed, the clinical trial could be penalized or completely shut down. Interim results are also of paramount importance to regulatory bodies to ensure data integrity and compliance with the priorly approved scientific and ethical standards [27,62,63].

Commitment to transparency is critical in fostering public trust and advancing scientific knowledge. A summary of all clinical trials, irrespective of the outcome, must be disclosed. A layperson summary needs to be handed in, which is a summary of the Clinical Study Report. Clinical trial sponsors are also required to provide the methodology of the trial in full detail, from administration route, dosage, and other medicine-related factors, up to the statistical analysis utilized for the selected patients. Generally speaking, commitment to open data sharing is also an encouragement to collaborate among researchers, allowing independent validation. The increased transparency not only helps the regulatory bodies, but it also helps to eliminate clinical trial failures and unnecessary repeat testing. From the patients’ perspective, transparent communication and data sharing enable patients to make informed decisions as they can assess the potential benefits and risks. The data and its transparency are vigorously monitored by the European Medicines Agency and national regulatory authorities.

Patients must be recipients of comprehensive and clear information about multiple aspects of the clinical trial objectives—what is the basis of their selection as possible candidates, risks, potential benefits, and alternatives—before agreeing to participate. All informed consent documents must be written in an accessible language, in the patient’s native tongue, where medical terms are simplified to ensure comprehension. The patient also has a right to ask medical personnel, and it must be ensured that the patient is not pressured into signing the consent forms and has enough time to consider participation. Special provisions are also included for vulnerable populations, such as pregnant or breastfeeding women, children, patients with cognitive impairments, and critically ill patients. Consent must also be gained from the mentioned populations, and children’s consent is also required (if capable), not only parents’. Participants have a right to withdraw consent at any time of the clinical trial without any repercussions. Adverse events and reactions must be immediately reported to regulatory authorities to enhance pharmacovigilance and patient safety. This real-time monitoring system ensures immediate intervention if it is necessary. Public representatives and patient advocacy groups are also included in the review process to ensure ethical considerations during the trial. If medically justified, the regulation also enforces post-trial obligations, where the patients have access to effective treatments after the trial concludes [45,46].

Clinical trials for medicinal products must undergo all phases; however, they can be categorized based on the level of risk posed to participants. Low-intervention trials involve investigational drugs that already possess approval for use, and where the safety profile is established, whilst high-risk trials include novel drug combinations, drugs, or new applications of existing data, where the safety data is limited. Risk assessment frameworks must be implemented in a structured manner to ensure that the high-risk trials receive greater scrutiny and regulatory oversight. The Regulation also encourages adaptive monitoring tailored to specific risks of the specific trials. On-site monitoring with medical professionals is necessary to ensure a rapid response to any emerging safety concerns. Remote monitoring or data review is usually used for low-risk trials, but it does not substitute the on-site standby by professionals. The utilization of telemedicine as wearable technology allows remote monitoring and real-time tracking of patient vitals. Additionally, the constant data flow allows better data management [58,65].

**Figure 2:**
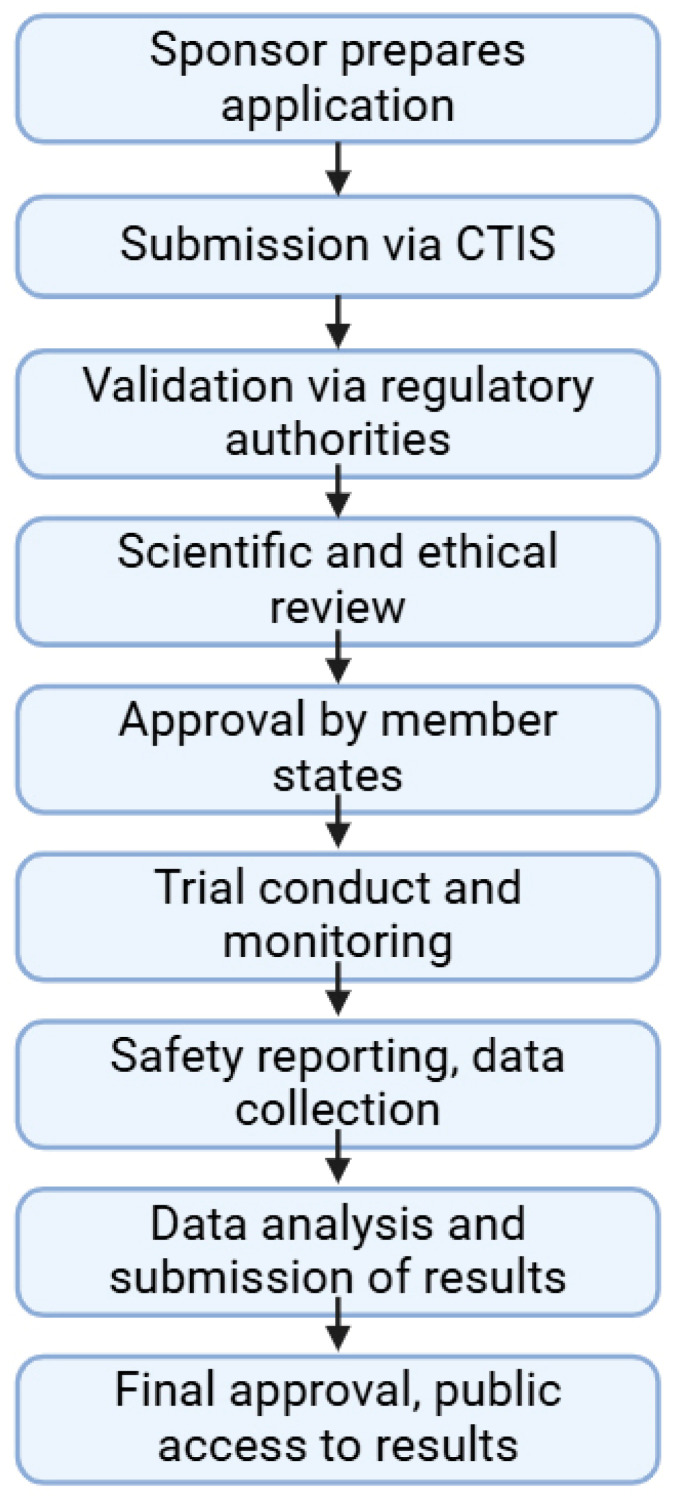
Schematics of the steps of clinical trials for medicinal products according to Clinical Trials Regulation No. 536/2014. Abbreviation used: CTIS—Clinical Trials Information System. Created in BioRender. Sipos, B. (2025) https://BioRender.com/qby0lpe.

There are multiple benefits to the implementation of Regulation 536/2014. For sponsors and researchers, it is claimed that it provides a simplified submission process, lower costs, and a faster time-to-market. The current evidence base does not support the claim that Regulation (EU) No 536/2014 has reduced the time-to-market and costs of clinical trials [66]. The Clinical Trials Information System (CTIS) aims to streamline processes under the regulation, but early experiences show mixed results. The European organization for Research and Treatment of Cancer (EORTC) conducted a study which revealed that regulatory comment response times averaged 27.5 days beyond the 12-day limit established by the regulation, thus indicating a need to enhance internal processes for new timeline compliance. Three multinational European clinical studies showed that approval timelines remain unacceptable despite some improvements because of conflicting application requirements and technical issues within CTIS. The regulation’s objectives are clear, but there is no concrete evidence to support reduced time-to-market and costs [67]. For patients, greater transparency and enhanced safety protections allow better compliance with the clinical trial, and they can access cutting-edge treatments. There are several challenges regarding Regulation 536/2014 as well—the transitioning from national systems to CTIS requires time and adjustment to national authorities. There is also a concern for data protection, where the increased transparency is to be balanced with GDPR compliance, as patients’ data must be held confidentially. A general structure of the authorization process of clinical trials for medicinal products can be seen in Figure 2.

## 5. Regulation of Clinical Trials for Medical Devices

The EU has also implemented stringent regulations for medical devices to ensure their performance, safety, and efficacy. The EU Medical Device Regulation (MDR) 2017/745 has replaced the prior Medical Device Directive (MDD) 93/42/EEC [44]. The main differences can be found in Table 2.

Regarding the regulations of clinical trials under the MDR, Articles No. 62–82 address their scientific, ethical, and authorization aspects. Article 62 outlines the fundamental requirements for conducting clinical trials. It is mandatory to comply with Good Clinical Practice, just like in medicinal products, to ensure safety and patient rights. Manufacturers may justify the necessity of clinical investigation, but only if there is sufficient data from previous products or trials. The study design must be robust, and a clear methodology and protocol must also be set up, especially for high-risk classified devices. The same principles apply regarding ethical considerations for medicinal products. Article 63 describes the necessity for informed consent, which is especially important for vulnerable populations, such as minors, and pregnant or breastfeeding women. The same rules apply, if minors are involved in the trial process, then their assent must also be obtained, in addition to parental consent. The mentioned vulnerable populations and their specifications are mentioned in Articles 65 to 67. The extension of the obtainment of an informed consent form is addressed in Article 64, where the principles of clinical investigations on incapacitated subjects are also considered. The rest of the articles describe the means to comply with the supervisory regulatory authorities [44].

First, manufacturers must submit a documentation pack to their relevant competent authority. This is mostly their national authority, but the documents must also be submitted to other members’ authorities if the trial overlaps multiple member states. The main documentation to be submitted is the Clinical Investigation Plan (CIP) which is a comprehensive document outlining the purpose of the research, the design of the device itself, and generic data regarding investigators and manufacturers. It should also include details and criteria on subject selection, risk management protocols, ethical considerations, data management, and evaluation methodology, amongst many others. The CIP documentation must be in full harmony with the GCP guidelines [47,63]. The investigator’s brochure is a detailed compilation of all preclinical and clinical data relevant to the device under investigation. Technical details of device design, mechanism of action, and possible adverse or beneficial effects can be found here. It is ensured that investigators have proper knowledge about the device on trial. A risk management plan is also to be submitted which should be in line with international standards, such as ISO 14971 guideline on the application of risk management to medical devices. General and similar documentation must also be submitted, such as ethical approvals from the ethical committee of the competent agency and the patient informed consent documents.

**Figure 3:**
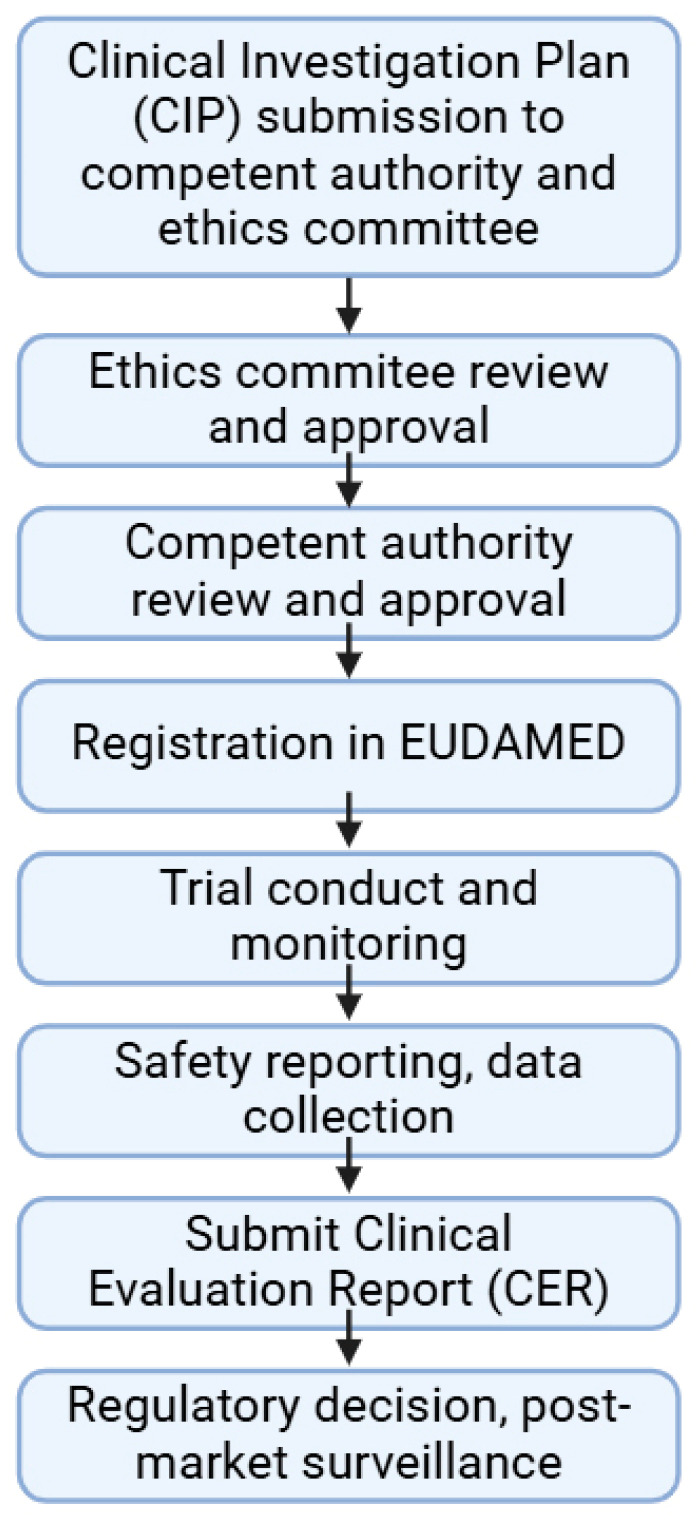
Schematics on the clinical trial authorization process of medical devices according to MDR 201/745. Abbreviations used: CIP—clinical investigation plan; CER—clinical evaluation report. Created in BioRender. Sipos, B. (2025) https://BioRender.com/r5ngnyr.

Ethical approval is also based on multiple factors, including the scientific evaluation of the proposed trial, its risk–benefit analysis, the qualifications of investigators, and finally, the assessment of the informed consent procedures and documentation. The average approval time lies between 60 and 90 days if all safety and regulatory requirements are met, followed by registration in the EUDAMED database to ensure publicly accessible data. The results of the trial must be collected together into a Clinical Evaluation Report (CER), which is a critical document according to the MDR. The structure of the report must be made in alignment with MDR Annex XIV Part A to ensure a systematic approach to clinical evaluation. The CER must include the clinical background of the medical device investigated from design characteristics, intended use and clinical applications. The statistical analysis must also be performed in a manner that it can be comparable with prior devices (if they exist). As it is a living document, all new data from post-market surveillance and adverse effects must be added into the document. A schematic on the authorization and clinical trial process can be found in Figure 3.

Similarly to medical devices, recent changes have been implemented in combined drug–device products. According to the EMA guideline on quality documentation for medicinal products when used with a medical device, three main groups can be detected according to the position and utilization of medical devices in the administration or preparation of drugs (integral drug–device combination) [68]. Medical devices, or some of them, can be an integral part of the product without the possibility of reusing, such as prefilled syringes. Co-packaged products (medicine with a co-packaged device) are another type of these products, and the ones called referenced devices also fall under this guideline. From the manufacturing side, both GMP and the ISO 13485 must be followed [56]. Active comparators or standard-of-care controls become the preferred choice when placebo controls prove unethical or impossible to implement, particularly for high-risk or invasive devices. Adaptive trial designs, together with Bayesian methods, enable researchers to preserve scientific validity through methods that reduce patient exposure to substandard treatments. Real-world evidence together with historical control data supports regulatory evaluations for accelerated approvals when randomization proves impractical. The evidence base becomes stronger through supplementary methods, which include simulation-based assessments and human factor studies and in silico modeling when direct comparisons are restricted. The process of bridging data and modular submissions enables the reduction in ethically challenging trials for devices that already hold CE-mark or previous validation. The process of ethical oversight demands continuous collaboration between researchers and ethics committees and regulatory authorities from the beginning. The new approaches in drug–device combination product evaluation focus on developing flexible methods that maintain ethical standards [69].

Understanding the primary mode of action (PMOA) is essential, as it depends on the composition of the combined drug–device product. This results in a dual regulation process, involving both CTR and MDR, emphasizing the necessary proof. Such an approach may shift the traditional sequence of clinical trials. For example, if a familiar active substance is used in a new administration device, attention may shift toward the performance of the device, while the safety and efficacy of the drug are emphasized only minimally. This leads to changes in risk assessment and evaluation, and in most cases, the low-risk-class devices are upgraded into a higher class due to the presence of the drug. Hybrid designs are applied with multiple factors included, and since drugs are involved, medical doctors must be included in the trial process. Investigators must be qualified in both drug administration and device use, and often multidisciplinary teams are included [70]. The timeline of the clinical trials now depend on their complexity, usually requiring more aspects in the clinical trial process to evaluate the quality and performance of the device, alongside the drug efficacy [71].

The EU MDR has transformed the regulatory landscape for medical devices. The regulations of clinical trials for medicinal products (drugs) have similar elements. With novel advancements in the field of devices or combined products, it is now recognized that all devices must be investigated and analyzed on (almost) the same principle as drugs. This is especially true for high-risk-classified medical devices and drug–device products. A comparative summary of the regulation of clinical trials can be found in Table 3.

## 6. Outlook of Non-EU Clinical Trials

### 6.1. Clinical Trials for Drugs

The UK has established its own clinical trials framework after Brexit because it no longer participates in the EU Clinical Trials Regulation (Regulation EU 536/2014) or the Clinical Trials Information System (CTIS). The Medicines and Healthcare products Regulatory Agency (MHRA) now receives EU clinical trial submissions directly instead of using CTIS for centralized coordination. The UK has created a combined assessment process which combines MHRA and Research Ethics Committees (RECs) through the Integrated Research Application System (IRAS). The UK government plans to establish a new regulatory framework after 2023 through a consultation process that will provide flexible risk-based trial classifications and expedited approval processes. The UK maintains equivalent requirements for trial registration and transparency but might implement flexible policies to boost innovation and market competitiveness. The UK maintains its acceptance of EU Clinical Trials Directive (2001/20/EC) and Good Clinical Practice (GCP) standards under ICH guidelines [63]. The EU provides CTIS coordinated assessment for multinational trials, but UK sponsors need to conduct separate assessments, which results in duplicated work for multi-country studies. The UK has launched support programs for decentralized and digital trials at a faster pace than the EU has achieved. The EU and UK maintain ongoing initiatives to achieve standardization in pharmacovigilance, but their systems operate independently. Sponsors who run parallel trials across the EU and UK must submit their applications and safety data and amendments to two independent systems. The UK’s flexible regulatory framework draws sponsors but creates additional complexity for trial operations. The EU and UK require separate regional approaches for clinical development strategies [72].

The Food and Drug Administration (FDA) operates under Title 21 of the Code of Federal Regulations (CFR) to govern drug clinical trials through Parts 50, 54, 56, and 312 and through the review of Investigational New Drug (IND) applications [73]. The EU follows Regulation (EU) 536/2014 for clinical trials, which is managed through CTIS and Member State coordination. One major distinction exists in submission procedures because the U.S. FDA needs one IND submission directly to the agency, while the EU system requires multiple stakeholders to review submissions through CTIS. The U.S. regulatory system provides faster processing of initial trial applications for early-phase studies due to its centralized nature. The FDA dedicates significant attention to pre-IND meetings and expedited pathways (Fast Track and Breakthrough Therapy) and adaptive designs. Both jurisdictions enforce GCP standards through ICH guidelines, but the U.S. regulatory framework provides more freedom to adapt trials and use interim data [74]. The FDA demonstrates more advanced capabilities in using real-world evidence and decentralized trial tools than the EU does since EU Member States show varying levels of adoption. Safety reporting regulations show distinction between the EU and U.S. because the EU requires EudraVigilance for SUSAR reporting across all Member States, but the U.S. uses the FDA Adverse Event Reporting System (FAERS) for direct FDA reporting. Both regulatory bodies mandate trial registration and result disclosure, butt EU CTIS demands automatic public access to most trial documents, whereas ClinicalTrials.gov in the U.S. lets sponsors choose what documents to redact. U.S. ethics oversight functions through Institutional Review Boards (IRBs) as a decentralized system, whereas the EU relies on national ethics committees that operate in conjunction with CTIS procedures. The definitions of “sponsor” and “serious adverse event” differ between the two regions, thus influencing compliance and documentation methods. The FDA’s process stands out for its centralized structure and dynamic nature, which benefits innovative therapeutic development [75].

China has experienced fast-paced changes in its clinical trial regulatory system since its membership in the ICH started in 2017. The National Medical Products Administration (NMPA) leads drug trial oversight and has introduced substantial changes to decrease review periods while following international standards [76]. The Chinese regulatory system grants automatic approval to clinical trial applications through a “silent approval” process when no objections are received within 60 working days. The Chinese ethics review process operates at the hospital or provincial level through Institutional Review Boards (IRBs), which results in inconsistent review times and interpretation methods. All submitted documents must be translated into Chinese for approval purposes in China, but the EU accepts English documents without additional requirements. The EU accepts multinational data with justifications for foreign drugs, but China demands local clinical trial data or bridging studies before approval unless a waiver is granted [76]. The GCP system in China follows ICH E6 standards, but some procedural aspects such as subject protection and inspection protocols have stricter enforcement. The EU uses CTIS to enhance transparency, but China has just started requiring trial registration on the Drug Clinical Trial Registry and Information Platform (DCTIRP). China’s pharmacovigilance system after approval has not reached the same level of maturity as EudraVigilance in the EU [77]. The Chinese regulatory system demonstrates strong state support for domestic innovation through its approach, but foreign sponsors encounter both additional scrutiny and strategic barriers. The differences between data integrity standards and site inspection procedures create challenges for conducting cross-regional trial operations. Sponsors need to develop specific strategies for China because its clinical and regulatory environment differs from other regions despite advancing regulatory convergence [77].

The Pharmaceuticals and Medical Devices Agency (PMDA), together with the Ministry of Health, Labor, and Welfare (MHLW), functions as the regulatory body for clinical trials in Japan. The Pharmaceuticals and Medical Devices Act (PMD Act) governs Japan’s clinical trial process while the Clinical Trials Act provides ethical oversight specifically for investigator-initiated studies. The Japanese application and review system operates independently from the EU’s CTIS because it requires direct PMDA consultation and local ethics review [78]. The pre-trial consultation process in Japan follows a detailed structure, which results in longer review periods than those in the EU. All regulatory documents need to be submitted in Japanese, which creates additional administrative challenges for multinational sponsors. Japan requires new drug applications (NDAs) to include local clinical data, which frequently necessitates conducting bridging studies with foreign trial results [79]. The EU accepts global data for approval when it fulfills ICH and EU-specific requirements. The Japanese regulatory framework strictly enforces GCP compliance because it places high importance on protecting patients and maintaining data integrity [63]. Japan requires extensive post-market surveillance with Early Post-Marketing Phase Vigilance (EPPV) as part of its system, which does not exist in the EU. The transparency requirements in Japan are less comprehensive than those specified in EU Regulation 536/2014, especially regarding the disclosure of trial documents and decisions to the public. The Japanese ethics review process varies based on trial type, but CTIS in the EU combines ethics and regulatory assessments. Japan maintains a cautious stance toward risk and innovation as an ICH member which results in delayed adoption of new trial approaches including decentralized trials. Japan and the EU maintain common regulatory fundamentals, but their operational frameworks and cultural regulatory approaches differ [78].

The Russian clinical trial landscape operates under the supervision of the Ministry of Health through the Federal Service for Surveillance in Healthcare (Roszdravnadzor). The Russian Clinical Trials Registry demands registration of clinical trials, and both local and international sponsors need prior authorization for their activities [80]. The Russian regulatory system operates with less transparency than the EU CTIS system because it restricts public access to trial documents and decision-making processes [18]. The Russian trial application process requires a centralized review that includes scientific, pharmacological, and ethical assessments, which extend the overall review duration. The state-appointed organization conducts a mandatory expert evaluation which differs from the EU’s sponsor-led submission process. All Russian clinical trials need to take place at sites that have received accreditation while following national GCP standards which match ICH requirements but contain unique local elements [63]. All submitted documents must be in Russian because of the substantial language barriers that exist. The EU allows multinational data acceptance, but Russia demands local population trials to study ethnic and demographic differences. The current geopolitical situation restricts international collaboration because sanctions together with regulatory isolation prevent data sharing and multi-country trial participation. The Russian post-market surveillance system lacks the advanced features of EudraVigilance because it provides minimal systematic signal detection capabilities and public-reporting functions. The transparency of clinical trials remains low, while sponsors need to overcome additional challenges when negotiating contracts, accessing data, and dealing with inspections. The Russian trial environment maintains fundamental clinical standards but operates with stricter rules, reduced transparency, and less global regulatory integration compared to the EU [81].

### 6.2. Clinical Trials for Medical Devices

Medical device regulation together with clinical trial governance have started to operate independently after the UK left the EU. The EU Medical Device Regulation (MDR, Regulation (EU) 2017/745) established detailed requirements for clinical evaluation and post-market surveillance, which apply most strongly to high-risk devices [82]. The EUDAMED system coordinates all clinical investigations, but its complete functionality remains under development. The UK maintains its UK Medical Devices Regulations 2002, which originated from the EU Medical Devices Directive (MDD), while it builds its own independent regulatory framework. The MHRA supervises device clinical trials in the UK, and IRAS serves as the platform for submitting applications [83]. The UK maintains its own regulatory approach because it does not use EUDAMED, so parallel strategies must be implemented for trials conducted throughout Europe. The UK ethical approval process functions through centralized review systems, which operate similarly to EU systems but lack CTIS-like coordination mechanisms. The UK authorities plan to implement international standards such as IMDRF and ISO 14155 while developing an innovation-friendly framework that includes enhanced digital and adaptive trial capabilities [84]. The UK maintains flexibility in its regulatory approach since it does not require clinical investigation for Class III and implantable devices as the EU MDR does. The EU has implemented more aggressive policies for phasing out MDD certificates than the UK has during its transition period. The MHRA requires UK manufacturers to obtain clinical investigation approvals independently, while EU sponsors need to work with Notified Bodies under MDR Article 62 when applicable [83]. The UK government established a new regulatory framework after Brexit which aims to simplify device trial procedures and speed up market entry processes. The current differences between EU and UK documentation requirements and classification rules and timelines force device sponsors to develop separate strategies for each market [85].

The U.S. FDA implements a risk-based strategy through streamlined pathways (e.g., 510(k), De Novo) whereas the EU’s MDR demands extensive clinical testing for high-risk medical devices. The FDA enables sponsors to depend on non-clinical data and predicate comparisons, but the EU has established stricter equivalence requirements. The FDA allows U.S. sponsors to conduct IDE trials for significant-risk studies under IRB supervision, but EU trials need ethics committee approval and national competent authority oversight through EUDAMED. The FDA provides formal pre-submission interactions through Q-Sub which occur more frequently than in the EU [86]. Real-world evidence serves as a more important factor for U.S. approval processes although the EU is transitioning to this approach through post-market clinical follow-up requirements [87]. The U.S. review process for clinical trials is faster because it operates under centralized oversight, whereas the EU needs multiple states to coordinate their reviews. The EU demands compliance with ISO 14155 standards, and MDR imposes more stringent post-market requirements. The EU has enhanced transparency in trial registration through EUDAMED, but ClinicalTrials.gov operates as a voluntary system in the United States. The U.S. regulatory pathways deliver more predictable and faster approvals for new technological products to manufacturers [86].

The Chinese regulatory process requires most imported devices to undergo local clinical trials or bridging studies, which presents stricter requirements than the EU MDR [88]. The clinical evaluation process in China, under the NMPA, requires additional local data, as foreign equivalence claims are not accepted. EU MDR offers detailed guidelines through Annex XIV for conducting coordinated assessments among all member states. China’s CMD performs reviews which adhere to strict administrative requirements and language protocols, consequently extending the review duration. EU sponsors benefit from standardized procedures through the EUDAMED system, which also enables future centralized registration. China does not have an equivalent centralized public trial registry system, and its operations lack transparency [88]. The two jurisdictions require compliance with ISO 14155 standards, yet Chinese organizations must follow domestic administrative rules during implementation [84]. China provides expedited approval for new medical devices that differ from EU MDR designation criteria. The requirements for post-market surveillance in China show ongoing development, yet they remain less sophisticated than the EU’s PMCF framework. The process of entering the Chinese market requires sponsors to conduct dedicated clinical trials, which extends both time and expense for global sponsors [89].

The Japanese PMDA regulates clinical trials for medical devices, but it shows less rigidity in accepting foreign data compared to EU MDR requirements [90]. Japan accepts both literature and equivalence data under particular conditions, although the EU has strengthened equivalence requirements mainly for Class III devices under MDR. ISO 14155 applies to both jurisdictions although Japan strictly enforces detailed documentation requirements together with Japanese-language compliance [91]. EU submissions experience higher levels of harmonization and coordinated review compared to Japan, which performs sequential and time-consuming evaluations. Ethical reviews in Japan occur at different locations across the country, whereas the EU uses EUDAMED for centralized management. Japan requires proof of safety and usability in the domestic market through the use of bridging studies [90]. The EU has a more transparent EUDAMED portal, but Japan provides less transparency for clinical trials. Both countries provide pre-submission advisory services, but Japan’s PMDA consultations function as formalized procedures. The systems employ different post-market monitoring structures, but they demonstrate strong robustness in both cases. Japan maintains a defensive approach that focuses on domestic needs, while the EU works to establish European-wide regulatory standards [91].

The Russian medical device trial management system operates through Roszdravnadzor as its central authority which demands domestic testing and approval from specialized expert committees. The Russian medical device regulations differ from EU MDR because they do not permit the acceptance of clinical trial data from foreign countries without local testing. Every submission needs to be in Russian language, with specific documentation requirements and Russian site locations for trial conduct. The EU provides better transparency through EUDAMED but enables multinational data acceptance when specific equivalence rules apply. The EU maintains a public trial registry, while Russia does not have this capability, and its post-market surveillance system remains underdeveloped compared to the EU’s mandatory PMCF. The Russian Federation recognizes ISO 14155, but its application remains inconsistent throughout the country. The EU regulatory framework delivers more harmonized reviews based on risk assessment, yet Russia prioritizes administrative compliance. The geopolitical environment along with limited recognition of clinical data between countries obstructs the development of global standards. EU trials enable better market reach and innovation preparedness, but Russia operates with localized and unclear processes. Global sponsors encounter substantial difficulties when attempting to match EU and Russian regulatory standards [92,93,94].

## 7. Conclusions

The European Union’s regulatory framework maintains distinct clinical evaluation procedures for medical devices and medicinal products because their mechanisms of action and risk profiles and intended uses differ. The Clinical Trials Regulation (EU) No 536/2014 establishes medicinal product regulation by requiring centralized trial authorization through the Clinical Trials Information System (CTIS). The system unifies EU Member State procedures for trials by implementing controlled multi-phase study designs that generate high-quality, statistically valid data to prove efficacy and safety. Medical devices undergo clinical investigations under the framework of Regulation (EU) 2017/745 which is known as MDR. EUDAMED presents a centralized framework for these trials although its full operational capabilities have not been achieved, and decentralization maintains its current oversight approach.

The main objectives of clinical trials stem from the fundamental scientific distinctions between these product categories. The therapeutic effect of an active substance requires drug trials to be established through defined endpoints across diverse population groups and different dosing regimens. Medical device trials focus on showing clinical performance and safety in specific device applications through methods that include pragmatic or observational designs together with real-world data collection. Under MDR, manufacturers must produce primary clinical data instead of using clinical equivalence claims, which has increased the requirements for new evidence. The transition to new standards proved difficult for both established medical devices and small- and medium-sized enterprises because new data generation does not generate immediate investment returns. The oversight systems and transparency measures differ substantially between these two regulatory sectors. The medicinal product trial system benefits from an existing ethical approval framework along with mandatory public registration requirements and standardized reporting deadlines. The current model of device trials shows ongoing efforts to improve transparency, but national approaches create inconsistencies in ethical review standards, trial registration, and result dissemination practices. EUDAMED’s complete implementation will achieve better alignment, but the main obstacle lies in its practical deployment across all EU nations and its integration with CTIS and other data systems.

Multiple regulatory enhancements are anticipated in the future. The EUDAMED system will establish standardized data reporting which enables better tracking and allows centralized access to device trial data as well as serious adverse event reports and post-market performance information. The system will create enhanced public trust and improve clinical and patient decision-making abilities. However, challenges persist. The integration of digital health tools and artificial intelligence and adaptive or self-learning systems creates new methodological and ethical problems regarding algorithm validation in time and patient data protection in connected systems. Regulatory science needs to develop specific trial approaches and evidence-generating methods for these emerging technologies. EU-specific requirements have the potential to create obstacles for innovative devices to enter the market because they differ from other jurisdictions’ regulatory frameworks. The inconsistency between ethics committee practices and MDR interpretation by Notified Bodies, together with prolonged clinical trial authorization across member states, creates efficiency challenges and higher costs which affect SMEs particularly. The regulatory burden from multiple compliance requirements across countries threatens to slow innovation because of inadequate streamlining of parallel obligations.

Regulatory convergence between medical device and pharmaceutical oversight for post-market surveillance and lifecycle monitoring and transparency purposes shows promising signs for long-term advantages despite current reservations. A complete harmonization of regulatory frameworks seems improbable, since fundamental differences between product nature and risk–benefit assessment paradigms exist. The successful navigation of this changing regulatory environment will require strong scientific advice systems together with early regulatory interactions and specific guidance for combination products and sustained investment in regulatory expertise. Manufacturers who understand product classification and regulatory pathways and evidence expectations can create efficient development programs that are both ethically sound and cost-effective for medical devices and pharmaceuticals.

## Figures and Tables

**Table 1:** Comparison of Clinical Trials Directive 2001/20/EC and the currently applied Clinical Trials Regulation NO. 536/2014. Abbreviation used: CTIS—Clinical Trials Information System.

Feature	Directive 2001/20/EC	Regulation No. 536/2014
Ethics Committee role	national discretion	in ordinance with all member states
Application Submission	separately to each member state	single application via CTIS
Assessment timeline	varying between member states	harmonized deadlines
Risk-based supervision	uniform requirements	based on risk level
transparency	limited public access	mandatory public access
Safety reporting	varying formats between member states	standardized

**Table 2:** Comparison of Medical Device Regulation 2017/745 and the prior Medical Device Directive 93/42/EEC. Abbreviations used: MDR—Medical Device Regulation; MDD—Medical Device Directive.

Aspect	MDR 2017/745	MDD 93/42/EEC
Legal framework	Binding regulation	Directive
Scope	Covers a broader range of devices	Gaps regarding certain products
Classification of devices	Stringent classification rules	Less strict classification rules
Clinical evidence requirements	Stronger requirements	Less stringent requirements
Post-market surveillance	Enhanced requirements	Basic requirements
Unique device identification (UDI)	Mandatory	Not mandatory
Notified bodies oversight	Stronger role and increased scrutiny	Less oversight and scrutiny
EUDAMED database	Fully integrated database with public access	Limited database and availability
Manufacturer responsibilities	Greater and stricter responsibilities	Fewer responsibilities
Transition period	The 3-year transition from May 2017	Transitioned out as MDR came into action

**Table 3:** Comparative summary of clinical trials for medicinal products (drugs), medical devices and combined drug–device products. Abbreviations: CTR—Clinical Trials Regulation; MDR—Medical Device Regulation; CTIS—Clinical Trials Information System; EMA—European Medicines Agency; RCT—Randomized controlled trials; GMP—Good Manufacturing Practice; ISO—International Organization for Standardization.

Aspect	Medicinal Product (Drug)	Medical Device	Combined Drug–Device Product
Regulation	Clinical Trials Regulation No. 536/2014	Medical Device Regulation 2017/745	Dual regulation: primarily CTR or MDR depending on primary model of action
Approval process	Clinical Trial Application via CTIS (regulatory and ethics approval)	Clinical investigation application with a submission to the competent authority and ethics committee	Coordinated submission based on the primary mode of action
Risk classification	No formal trial-based classification, as all investigational drugs must undergo a strict review	Classified as I, IIa, IIb, and III based on invasiveness and risk	Risk driven by both drug safety profile and device classification (usually elevated)
Clinical trial phases	Following Phase I–IV	Follows feasibility and pivotal studies	May include hybrid trial phases combining tests for drug efficacy and device performance
Study design	Randomized controlled trials	Flexible, comparative studies	RCTs with embedded device usability/performance metrics
Sample size	Hundreds to thousands of patients	Dozens to hundreds	Depends on both drug efficacy and device-specific endpoints, but larger than for medical devices
Clinical endpoint	Pharmacokinetics, efficacy, and safety	Device performance, usability, functionality	Based on the assessment of both drug and medical device performance
Regulatory review time	12–18 months until EMA approval	Faster for low-risk devices, whilst Class III requires rigorous notified body assessments	Can be prolonged due to dual evaluation
Post-market surveillance	Adverse drug reaction monitoring	Device failure and usability issues monitoring	Requires integrated vigilance system covering both adverse drug reactions and device incidents
Manufacturing requirements	Good Manufacturing Practice	ISO 13485 (Medical Device Quality Management System) [56]	Must comply with both GMP and ISO 13485 [56]
Investigator requirements	Medical doctor	Healthcare professional	Medical doctor
Interaction with the human body	Usually works systematically	Usually works locally	Usually combined systemic and localized effects
Need for placebo control	Standard	Rare or unethical	Standard for the drug part, but ethically it should be justified for the device part

## Data Availability

All data are available upon request from the corresponding author.

## Abbreviations

The following abbreviations are used in this manuscript:

CER	Clinical Evaluation Report
CIP	Clinical Investigation Plan
CTIS	Clinical Trials Information System
EU	European Union
GCP	Good Clinical Practice
GDPR	General Data Protection Regulation
GMP	Good Manufacturing Practice
ICH	The International Council for Harmonization of Technical Requirements for Pharmaceuticals for Human Use
ISO	International Organization for Standardization
MDD	Medical Device Directive
MDR	Medical Device Regulation
R&D	Research and development
RCT	Randomized controlled trial

## References

[1] Asgardoon M.H., Amirzade-Iranaq M.H., Mehri A., Piri S.M., Jalali P., Ghodsi Z., Dehghan H.R., Rahimi-Movaghar V., Salamati P. (2022). Adverse Impacts of Imposing International Economic Sanctions on Health. Arch. Iran. Med..

[2] Sawyer T., Gray M.M., Umoren R. (2022). The Global Healthcare Simulation Economy: A Scoping Review. Cureus.

[3] Mahlangu J. (2024). Emicizumab and Unmet Needs of Patients with Hemophilia a Who Are Managed with Replacement Therapies. Expert Rev. Hematol..

[4] DeGroot L., Pavlovic N., Perrin N., Dy S.M., Szanton S., Gilotra N., Denfeld Q., Miller H., McIlvennan C., Davidson P. (2024). The Impact of Unmet Palliative Care Needs and Physical Frailty on Clinical Outcomes: A Prospective Study of Adults with Heart Failure. J. Card. Fail..

[5] Demirci E., Knicley J., Fiorentino L. (2024). Clinical Development and Marketing Application Review Times for Novel Orphan-Designated Drugs. Front. Med..

[6] Kalland M.E., Pose-Boirazian T., Palomo G.M., Naumann-Winter F., Costa E., Matusevicius D., Duarte D.M., Malikova E., Vitezic D., Larsson K. (2024). Advancing Rare Disease Treatment: EMA’s Decade-Long Insights into Engineered Adoptive Cell Therapy for Rare Cancers and Orphan Designation. Gene Ther..

[7] Bouwman L., Sepodes B., Leufkens H., Torre C. (2024). Trends in Orphan Medicinal Products Approvals in the European Union between 2010–2022. Orphanet J. Rare Dis..

[8] Holmes D.R., Geoffrion R., Hunt J., Hance R.C., Leon M.B., Mack M.J., Kaplan A.V. (2022). Regulatory Strategies for Early Device Development and Approval. Catheter. Cardiovasc. Interv..

[9] Kallio M.J., Starokozhko V., Agricola E., Burggraf M., Heß A., Ballensiefen W., Löbker W., Nuevo Y., Pasmooij A.M.G., Mol P.G.M. (2023). Translating Academic Drug Discovery into Clinical Development: A Survey of the Awareness of Regulatory Support and Requirements Among Stakeholders in Europe. Clin. Pharmacol. Ther..

[10] Farlow A., Torreele E., Gray G., Ruxrungtham K., Rees H., Prasad S., Gomez C., Sall A., Magalhães J., Olliaro P. (2023). The Future of Epidemic and Pandemic Vaccines to Serve Global Public Health Needs. Vaccines.

[11] Pettitt D., Arshad Z., Davies B., Smith J., French A., Cole D., Bure K., Dopson S., DiGiusto D., Karp J. (2017). An Assessment of the Factors Affecting the Commercialization of Cell-Based Therapeutics: A Systematic Review Protocol. Syst. Rev..

[12] Kokudeva M., Vichev M., Naseva E., Miteva D.G., Velikova T. (2024). Artificial Intelligence as a Tool in Drug Discovery and Development. World J. Exp. Med..

[13] Zhang Y., Mastouri M., Zhang Y. (2024). Accelerating Drug Discovery, Development, and Clinical Trials by Artificial Intelligence. Med.

[14] Rowan N.J. (2024). Digital Technologies to Unlock Safe and Sustainable Opportunities for Medical Device and Healthcare Sectors with a Focus on the Combined Use of Digital Twin and Extended Reality Applications: A Review. Sci. Total Environ..

[15] Hasselgren C., Oprea T.I. (2024). Artificial Intelligence for Drug Discovery: Are We There Yet?. Annu. Rev. Pharmacol. Toxicol..

[16] Pfob A., Hillen C., Seitz K., Griewing S., Becker S., Bayer C., Wagner U., Fasching P., Wallwiener M., Kommission Digitale Medizin, Deutsche Gesellschaft für Gynäkologie und Gebursthilfe (DGGG) (2024). Status Quo and Future Directions of Digitalization in Gynecology and Obstetrics in Germany: A Survey of the Commission Digital Medicine of the German Society for Gynecology and Obstetrics. Arch. Gynecol. Obstet..

[17] Knitza J., Gupta L., Hügle T. (2024). Rheumatology in the Digital Health Era: Status Quo and Quo Vadis?. Nat. Rev. Rheumatol..

[18] Kandi V., Vadakedath S. (2023). Clinical Trials and Clinical Research: A Comprehensive Review. Cureus.

[19] Goetz L.H., Schork N.J. (2018). Personalized Medicine: Motivation, Challenges, and Progress. Fertil. Steril..

[20] Makurvet F.D. (2021). Biologics vs. Small Molecules: Drug Costs and Patient Access. Med. Drug Discov..

[21] Wouters O.J., Vogel M., Feldman W.B., Beall R.F., Kesselheim A.S., Tu S.S. (2024). Differential Legal Protections for Biologics vs Small-Molecule Drugs in the US. JAMA.

[22] Weintraub W.S., Lee K.H. (2018). Advances in Cardiovascular Care. JACC Basic Transl. Sci..

[23] Maldonado V.V., Patel N.H., Smith E.E., Barnes C.L., Gustafson M.P., Rao R.R., Samsonraj R.M. (2023). Clinical Utility of Mesenchymal Stem/Stromal Cells in Regenerative Medicine and Cellular Therapy. J. Biol. Eng..

[24] Qiu T., Wang Y., Liang S., Han R., Toumi M. (2021). Partnership Agreements for Regenerative Medicines: A Database Analysis and Implications for Future Innovation. Regen. Med..

[25] Hofstädter-Thalmann E., Largier D. (2022). EFPIA Guideline on a Quality Framework of Principles in Lifelong Learning in Healthcare. J. Eur. CME.

[26] Herrero-Martinez E., Hussain N., Roux N.L., MacDonald J., Mayer M., Palacios R., Kühler T.C. (2022). Dynamic Regulatory Assessment: Evolving the European Regulatory Framework for the Benefit of Patients and Public Health—An EFPIA View. Clin. Ther..

[27] Andrianov A.K. (2023). Noncovalent PEGylation of Protein and Peptide Therapeutics. Wiley Interdiscip. Rev. Nanomed. Nanobiotechnol..

[28] Årdal C., Baraldi E., Theuretzbacher U., Outterson K., Plahte J., Ciabuschi F., Røttingen J.-A. (2018). Insights into Early Stage of Antibiotic Development in Small- and Medium-Sized Enterprises: A Survey of Targets, Costs, and Durations. J. Pharm. Policy Pract..

[29] Mulcahy A., Rennane S., Schwam D., Dickerson R., Baker L., Shetty K. (2025). Use of Clinical Trial Characteristics to Estimate Costs of New Drug Development. JAMA Netw. Open.

[30] Ngum N., Ndomondo-Sigonda M., Habonimana R., Siyoi F., Irasabwa C., Ojukwu J., Apolinary F., Okello A., Ahmada S., Walker S. (2024). Evaluation of the Review Models and Approval Timelines of Authorities Participating in the East African Medicine Regulatory Harmonisation Initiative: Alignment and Strategies for Moving Forward. Front. Med..

[31] MacPherson A., Hutchinson N., Schneider O., Oliviero E., Feldhake E., Ouimet C., Sheng J., Awan F., Wang C., Papenburg J. (2021). Probability of Success and Timelines for the Development of Vaccines for Emerging and Reemerged Viral Infectious Diseases. Ann. Intern. Med..

[32] Subramaniam D., Anderson-Smits C., Rubinstein R., Thai S.T., Purcell R., Girman C. (2024). A Framework for the Use and Likelihood of Regulatory Acceptance of Single-Arm Trials. Ther. Innov. Regul. Sci..

[33] Boyle J.R., Mani K. (2024). Improving Post-Market Surveillance for New Endovascular Devices. Eur. J. Vasc. Endovasc. Surg..

[34] Exner H.M., Gregorchuk B.S.J., Castor A.-G., Crisostomo L., Kolsun K., Giesbrecht S., Dust K., Alexander D.C., Bolaji A., Quill Z. (2024). Post-Market Surveillance of Six COVID-19 Point-of-Care Tests Using Pre-Omicron and Omicron SARS-CoV-2 Variants. Microbiol. Spectr..

[35] Triantafyllidi E., Triantafillidis J.K. (2022). Systematic Review on the Use of Biosimilars of Trastuzumab in HER2+ Breast Cancer. Biomedicines.

[36] Bischoff H., O’Connor N.K., Kim J., Popescu B.V., Bigot C., Pradhan S., Chakraborty R., Jaison L., Majeed F., Park L.S. (2025). Comparative Preclinical Evaluation of Tuznue Versus Referent Herceptin: A Registered Trastuzumab Biosimilar. Drugs R D.

[37] Garattini S., Natsis Y., Banzi R. (2021). Pharmaceutical Strategy for Europe: Reflections on Public Health-Driven Drug Development, Regulation, and Policies. Front. Pharmacol..

[38] Geiger S., Bourgeron T. (2023). In the Name of Transparency: Organizing European Pharmaceutical Markets through Struggles over Transparency Devices. Organ. Stud..

[39] Hemmerling T.M., Hofer I.S. (2022). Protecting Intellectual Property While Satisfying Scientific Transparency. Anesth. Analg..

[40] Gilbert P., Fawcett R., Coles J., Hillson W. (2022). Intellectual Property Rights and Vaccines. Methods Mol. Biol..

[41] Gaspar R.S., Silva-Lima B., Magro F., Alcobia A., da Costa F.L., Feio J. (2020). Non-Biological Complex Drugs (NBCDs): Complex Pharmaceuticals in Need of Individual Robust Clinical Assessment Before Any Therapeutic Equivalence Decision. Front. Med..

[42] Liu Y.-H., Chen Y.-S., Tseng T., Jiang M.-L., Gau C.-S., Chang L.-C. (2023). Regulatory Considerations for Generic Products of Non-Biological Complex Drugs. J. Food Drug Anal..

[43] Malheiro V., Duarte J., Veiga F., Mascarenhas-Melo F. (2023). Exploiting Pharma 4.0 Technologies in the Non-Biological Complex Drugs Manufacturing: Innovations and Implications. Pharmaceutics.

[44] Regulation (EU) 2017/745 of the European Parliament and of the Council of 5 April 2017 on Medical Devices, Amending Directive 2001/83/EC, Regulation (EC) No 178/2002 and Regulation (EC) No 1223/2009 and Repealing Council Directives 90/385/EEC and 93/42/EEC 2017. https://eur-lex.europa.eu/legal-content/EN/TXT/PDF/?uri=CELEX:32017R0745.

[45] Schmitz A.A., Font-Nieves M., Doucouré T., Podhaisky H.-P. (2025). Impact of Rule 11 on the European Medical Software Landscape: Analysis of EUDAMED and Further Databases Three Years After MDR Implementation. Ther. Innov. Regul. Sci..

[46] Antal A., Ganho-Ávila A., Assecondi S., Barbour T., Bjekić J., Blumberger D.M., Bolognini N., Brunelin J., Chanes L., Dale M. (2024). The Consequences of the New European Reclassification of Non-Invasive Brain Stimulation Devices and the Medical Device Regulations Pose an Existential Threat to Research and Treatment: An Invited Opinion Paper. Clin. Neurophysiol..

[47] (2019). Medical Devices—Application of Risk Management to Medical Devices.

[48] Maci J., Marešová P. (2022). Critical Factors and Economic Methods for Regulatory Impact Assessment in the Medical Device Industry. Risk Manag. Healthc. Policy.

[49] Xu M., Zhang L., Feng X., Zhang Z., Huang Y. (2022). Regulatory Reliance for Convergence and Harmonisation in the Medical Device Space in Asia-Pacific. BMJ Glob. Health.

[50] Huusko J., Kinnunen U.-M., Saranto K. (2023). Medical Device Regulation (MDR) in Health Technology Enterprises—Perspectives of Managers and Regulatory Professionals. BMC Health Serv. Res..

[51] Beddoe-Rosendo J., Heaysman C.L., Hajnal J.V., Ourselin S., Vanhoestenberghe A. (2023). Medical Device Regulatory Challenges in the UK Are Affecting Innovation and Its Potential Benefits. Proc. Inst. Mech. Eng. H.

[52] Petrick N., Chen W., Delfino J.G., Gallas B.D., Kang Y., Krainak D., Sahiner B., Samala R.K. (2023). Regulatory Considerations for Medical Imaging AI/ML Devices in the United States: Concepts and Challenges. J. Med. Imaging.

[53] Aronson J.K., Heneghan C., Ferner R.E. (2020). Medical Devices: Definition, Classification, and Regulatory Implications. Drug Saf..

[54] Matovu B., Baluka J.W., Takuwa M., Namuli L.K., Mpaata C.N., Mugaga J., Mulindwa B., Nalwoga R., Wolters M.K., Ssekitoleko R.T. (2023). Translating Medical Device Innovations to Market—A Ugandan Perspective. BMC Res. Notes.

[55] Bretthauer M., Gerke S., Hassan C., Ahmad O.F., Mori Y. (2023). The New European Medical Device Regulation: Balancing Innovation and Patient Safety. Ann. Intern. Med..

[56] (2016). Medical Devices—Quality Management Systems—Requirements for Regulatory Purposes.

[57] Kidholm K., Jensen L.K., Johansson M., Montori V.M. (2023). Telemedicine and the Assessment of Clinician Time: A Scoping Review. Int. J. Technol. Assess. Health Care.

[58] Fields B.G. (2020). Regulatory, Legal, and Ethical Considerations of Telemedicine. Sleep Med. Clin..

[59] Solimini R., Busardò F.P., Gibelli F., Sirignano A., Ricci G. (2021). Ethical and Legal Challenges of Telemedicine in the Era of the COVID-19 Pandemic. Medicina.

[60] Rowe S.P., Pomper M.G. (2022). Molecular Imaging in Oncology: Current Impact and Future Directions. CA Cancer J. Clin..

[61] Rodriguez-Manzano J., Subramaniam S., Uchea C., Szostak-Lipowicz K.M., Freeman J., Rauch M., Tinto H., Zar H.J., D’Alessandro U., Holmes A.H. (2024). Innovative Diagnostic Technologies: Navigating Regulatory Frameworks through Advances, Challenges, and Future Prospects. Lancet Digit. Health.

[62] European Parliament and Council of the European Union (2014). Regulation (EU) No 536/2014 of the European Parliament and of the Council of 16 April 2014 on Clinical Trials on Medicinal Products for Human Use, and Repealing Directive 2001/20/EC Text with EEA relevance. Off. J. Eur. Union.

[63] International Council for Harmonisation of Technical Requirements for Pharmaceuticals for Human Use (ICH) (2016). ICH Guideline for Good Clinical Practice E6(R2).

[64] Patrick-Brown T.D.J.H., Bourner J., Kali S., Trøseid M., Yazdanpanah Y., Olliaro P., Olsen I.C. (2024). Experiences and Challenges with the New European Clinical Trials Regulation. Trials.

[65] Yamada O., Chiu S.-W., Takata M., Abe M., Shoji M., Kyotani E., Endo C., Shimada M., Tamura Y., Yamaguchi T. (2021). Clinical Trial Monitoring Effectiveness: Remote Risk-Based Monitoring versus on-Site Monitoring with 100% Source Data Verification. Clin. Trials.

[66] Gnanasakthy A., Levy C., Norcross L., Doward L., Winnette R. (2023). A Review of Labeling Based on Patient-Reported Outcome Endpoints for New Oncology Drugs Approved by the European Medicines Agency (2017–2021). Value Health.

[67] Stans J., Verbandt S., Kromar S., Deleersnijder A. (2023). Implementation of Regulation (EU) No 536/2014 as a Non-Commercial Sponsor: An Internal Survey and a Descriptive Analysis of Timelines. Ther. Innov. Regul. Sci..

[68] (2021). Guideline on Quality Documentation for Medicinal Products When Used with a Medical Device.

[69] Bianchini E., Mayer C.C. (2022). Medical Device Regulation: Should We Care About It?. Artery Res..

[70] Beckers R., Kwade Z., Zanca F. (2021). The EU Medical Device Regulation: Implications for Artificial Intelligence-Based Medical Device Software in Medical Physics. Phys. Med..

[71] Kumoluyi R., Khanolkar A. (2022). Risk Management in Drug-Device Combination Product Development. Ther. Innov. Regul. Sci..

[72] Hofer M.P., Criscuolo P., Shah N., Wal A.L.J.T., Barlow J. (2022). Regulatory Policy and Pharmaceutical Innovation in the United Kingdom after Brexit: Initial Insights. Front. Med..

[73] Corbin J., Walker A.J. (2023). FDA Overview. Translational Radiation Oncology.

[74] Ramamoorthy A., Araojo R., Vasisht K.P., Fienkeng M., Green D.J., Madabushi R. (2023). Promoting Clinical Trial Diversity: A Highlight of Select US FDA Initiatives. Clin. Pharmacol. Ther..

[75] Turner J.R. (2020). New FDA Guidance on General Clinical Trial Conduct in the Era of COVID-19. Ther. Innov. Regul. Sci..

[76] Li S., Li J., Zeng W., Li Z., Zhang J., Wang S., Xu N., Li Z. (2025). Re-Analysis of the Current Status of Clinical Trial Registration in China. Front. Med..

[77] Zhou Q., Chen X.-Y., Yang Z.-M., Wu Y.-L. (2017). The Changing Landscape of Clinical Trial and Approval Processes in China. Nat. Rev. Clin. Oncol..

[78] Noguchi A., Hanaoka H., Uyama Y. (2022). Potential Future Drug Development Lag in Japan Based on an Analysis of Multiregional Clinical Trials in the US, Europe, and East Asia. Ther. Innov. Regul. Sci..

[79] Eba J., Nakamura K. (2022). Overview of the Ethical Guidelines for Medical and Biological Research Involving Human Subjects in Japan. Jpn. J. Clin. Oncol..

[80] Martynova E., Shcherbovich A. (2024). Digital Transformation in Russia: Turning from a Service Model to Ensuring Technological Sovereignty. Comput. Law Secur. Rev..

[81] Manoharan K., Jinson J., Ramesh K., George M. (2023). Clinical Trial Trends over the Last 5 Years among the BRICS (Brazil, Russia, India, China, and South Africa) Nations. Perspect. Clin. Res..

[82] Fraser A.G., Redberg R.F., Melvin T. (2025). The Origins of Regulations for Pharmaceutical Products and Medical Devices—What Can Be Learned for the Governance of Medical Devices in Europe?. Eur. Rev..

[83] Ali F., Nollet L.M.L. (2025). Global Regulations of Medicinal, Pharmaceutical, and Food Products.

[84] (2020). Clinical Investigation of Medical Devices for Human Subjects—Good Clinical Practice.

[85] Curle A.J., Barnes J.L., Owen R., Barker R.A., El Haj A., Forbes S.J., Ghevaert C., Oreffo R.O., Rose F.R., Stevens M.M. (2024). A Decade of Progress: Achievements and Future Challenges for Regenerative Medicine Research in the United Kingdom. J. Immunol. Regen. Med..

[86] Muralidharan V., Adewale B.A., Huang C.J., Nta M.T., Ademiju P.O., Pathmarajah P., Hang M.K., Adesanya O., Abdullateef R.O., Babatunde A.O. (2024). A Scoping Review of Reporting Gaps in FDA-Approved AI Medical Devices. npj Digit. Med..

[87] Tettey F., Parupelli S.K., Desai S. (2024). A Review of Biomedical Devices: Classification, Regulatory Guidelines, Human Factors, Software as a Medical Device, and Cybersecurity. Biomed. Mater. Devices.

[88] Han Y., Bergmann J. (2024). Transforming Medical Regulations into Numbers: Vectorizing a Decade of Medical Device Regulatory Shifts in the USA, EU, and China. arXiv.

[89] Liao X., Yao C., Jin F., Zhang J., Liu L. (2024). Barriers and Facilitators to Implementing Imaging-Based Diagnostic Artificial Intelligence-Assisted Decision-Making Software in Hospitals in China: A Qualitative Study Using the Updated Consolidated Framework for Implementation Research. BMJ Open.

[90] Miyazawa M., Tanaka M., Tanaka Y., Terashima R., Ezura M., Miyazawa H., Ikuma M., Tomita Y. (2025). Concordance Between Pharmaceuticals and Medical Devices Agency Review and Ministry of Health, Labour and Welfare Decision Among New Drug Applications in Japan. Clin. Pharmacol. Ther..

[91] Chettri B., Ravi R. (2024). A Comparative Study of Medical Device Regulation between Countries Based on Their Economies. Expert Rev. Med. Devices.

[92] Gross M.L. (2025). Medical Sanctions Against Russia: Arresting Aggression or Abrogating Healthcare Rights?. Am. J. Bioeth..

[93] Pogarskaya A.S. (2024). On the Issue of Parallel Import of Medical Articles and Component Units to Them in the Russian Federation in Conditions of Sanctions Policy. Probl. Sotsialnoi Gig. Zdravookhranenniiai Istor. Med..

[94] Pawar H., Patel M. (2025). Harmonization of Regulatory Frameworks for Medical Devices in BRICS Countries: A Path to Enhanced Trade and Investment. Ann. Pharm. Françaises.

